# The Peptide Salamandrin-I Modulates Components Involved in Pyroptosis and Induces Cell Death in Human Leukemia Cell Line HL-60

**DOI:** 10.3390/pharmaceutics15071864

**Published:** 2023-07-01

**Authors:** Amandda Évelin Silva-Carvalho, Nakaly Natiely de Oliveira, Julia Viana Lafetá Machado, Daniel Carneiro Moreira, Guilherme Dotto Brand, José Roberto S. A. Leite, Alexandra Plácido, Peter Eaton, Felipe Saldanha-Araujo

**Affiliations:** 1 Laboratory of Hematology and Stem Cells (LHCT), Faculty of Health Sciences, University of Brasilia, Campus Darcy Ribeiro SN, Brasilia 70910-900, Brazil; amanddaevelin@hotmail.com (A.É.S.-C.); nakalynatiely@gmail.com (N.N.d.O.); julia_lafeta@hotmail.com (J.V.L.M.); 2 Research Center in Morphology and Applied Immunology, NuPMIA, Faculty of Medicine, University of Brasilia, Campus Darcy Ribeiro SN, Brasilia 70910-900, Brazil; moreiradc@unb.br (D.C.M.); jrsaleite@gmail.com (J.R.S.A.L.); 3 Institute of Chemistry, University of Brasilia, Campus Darcy Ribeiro SN, Brasilia 70910-900, Brazil; gdbrand@gmail.com; 4 LAQV/REQUIMTE, Department of Chemistry and Biochemistry, Faculty of Sciences, University of Porto, 4169-007 Porto, Portugal; 5 The Bridge, School of Chemistry, University of Lincoln, Lincoln LN6 7TS, UK; peaton@lincoln.ac.uk

**Keywords:** antioxidant peptide, salamandrin-I, leukemia, HL-60, pyroptosis

## Abstract

Amphibian secretions have been extensively investigated for the production of bioactive molecules. Salamandrin-I is an antioxidant peptide, isolated from the skin secretion of the fire salamander, that has induced no toxicity in microglia or erythrocytes. Importantly, the administration of antioxidants may constitute an adequate therapeutic approach to cancer treatment. Here, with the purpose of better characterizing the therapeutic potential of salamandrin-I, we investigated whether this antioxidant peptide also exerts anticancer activity, using the human leukemia cell line HL-60 as a cancer model. Salamandrin-I treatment induced a significant reduction in HL-60 proliferation, which was accompanied by cell cycle arrest. Furthermore, the peptide-induced cell death showed a significant increase in the LDH release in HL-60 cells. The cellular toxicity exerted by salamandrin-I is possibly related to pyroptosis, since the HL-60 cells showed loss of mitochondrial membrane potential and hyperexpression of inflammasome components following the peptide treatment. This is the first demonstration of the anticancer potential of the salamandrin-I peptide. Such results are important, as they offer relevant insights into the field of cancer therapy and allow the design of future bioactive molecules using salamandrin-I as a template.

## 1. Introduction

Salamanders are the second most diverse lineage of amphibians, with more than 760 living species. These amphibians have been used as models for scientific development in the most diverse fields of experimental biology. Importantly, they have served as classic models in the development of scientific research in the field of regenerative medicine due to their recognized ability to regenerate tissues, such as limbs, the heart, the spinal cord, lenses, and others. Furthermore, for hundreds of years, salamanders have served as a basis for the study of developmental biology because of the ease of access to their embryos. Additionally, salamanders have also been explored as important scientific models for studies of terrestrial locomotion, feeding, and toxicity [1,2].

As a defense mechanism against predators, salamanders are capable of producing a poisonous secretion through epidermal glands. Salamander venom has a diverse composition, but the presence of steroidal alkaloids of the salamandrine type stands out. Amphibian secretions have been extensively investigated for the production of other bioactive molecules that have important antimicrobial, anti-inflammatory, and antidiabetic properties [3,4,5]. In large part, the biological properties promoted by amphibian secretion are attributed to the presence of bioactive peptides, which include brevinins, bombesins, dermaseptins, esculentins, magainins, temporins, tigerinins, and salamandrins [6,7].

Recently, Plácido et al. isolated the salamandrin-I peptide from the skin secretion of the fire salamander (*Salamandra salamandra*). This peptide did not show antimicrobial activity against Gram-positive or -negative bacteria, did not promote in vitro cytotoxicity in microglia or erythrocytes, and did not show toxic effects in vivo when in contact with *Galleria mellonella* larvae. Interestingly, the authors of that study demonstrated that salamandrin-I showed antioxidant potential by scavenging DPPH and ABTS radicals [7]. Importantly, the excess of reactive oxygen species (ROSs) is associated with the pathogeneses of heart disease, neurodegenerative diseases, diabetes mellitus, and cancer [8,9,10,11]. Oxidative stress and excessive ROS production also seem to influence the pathogeneses of several hematological malignancies, such as myelodysplastic syndrome (MDS) [12], acute lymphoblastic leukemia (ALL) [13], and acute myeloid leukemia (AML) [14,15].

AML is the most common leukemia observed among the adult population, accounting for more than 80,000 deaths worldwide. It is characterized by the uncontrolled proliferation of myeloid blasts with impaired differentiation, resulting in ineffective hematopoiesis and life-threatening cytopenia. Cytogenetic abnormalities are also common in AML and have been used as criteria to define specific molecular subgroups [16,17]. The HL-60 cell line was derived from peripheral blood leucocytes obtained from a female adult patient who was initially diagnosed with acute promyelocytic leukemia (APL), a subtype of AML. APL is characterized by the presence of the PML/RARA gene, resulting from a reciprocal translocation, t(15;17), and leading to the fusion of the promyelocytic leukemia (PML) gene with the retinoic acid receptor alpha (RARA) gene. Additional karyotypic analysis of the HL-60 cell line revealed the absence of the t(15;17) translocation, leading to the classification of this cell line as AML M2. HL-60 is the most studied human acute myeloid leukemia cell line, representing a useful model for studying the processes of cell proliferation, death, and differentiation [18,19,20].

Oxidative stress has been recognized as an intrinsic feature of AML relapse [14]. Primary blasts from AML patients seem to produce high ROS levels associated with impaired antioxidant defenses, contributing to blast proliferation [15]. Interestingly, azelaic acid treatment was able to suppress proliferation and induce apoptosis and cell cycle arrest in AML patient cells and AML cell lines by decreasing ROS levels and increasing GSH and SOD levels [21]. Such observations suggest that the administration of antioxidants may constitute an adequate therapeutic approach for the treatment of these pathologies [22]. Similar findings have been observed in nonhematological cancers. Antioxidant peptides isolated from some oysters species were able to induce apoptosis in prostate, colon, and oral cancer cell lines, demonstrating that—in addition to scavenging free radicals—these compounds may also bear anticancer potential [23,24,25].

Here, with the purpose of better characterizing the therapeutic potential of salamandrin-I, we have investigated whether this antioxidant peptide also exerts anticancer activity, using the HL-60 human leukemia cell line as a cancer model.

## 2. Materials and Methods

### 2.1. Peptide Isolation and Cell Culture

The salamandrin-I peptide was isolated as has been previously described [7], and its characterization is shown in Supplementary Figure S1. The peptide used in this study was 95% pure, and it was solubilized in DMSO (Sigma Aldrich, St. Louis, MO, USA) prior to dilution in a Roswell Park Memorial Institute medium (RPMI 1640, Gibco Waltham, MA, USA) for use in all experiments. The HL-60 cell line—a promyelocytic cell line derived from human leukemia—was kindly provided by Prof. João Agostinho Machado-Neto (University of São Paulo, São Paulo, Brazil). The cells were maintained in an RPMI 1640 medium supplemented with 10% FBS (Gibco, Waltham, MA, USA), plus 1% penicillin/streptomycin (Sigma-Aldrich, St. Louis, MO, USA). The cell cultures were maintained at 5% CO_2_ and 37 °C, and the culture medium was replaced every 48 h.

### 2.2. MTT Assay

The effect of salamandrin-I treatment on HL-60 growth (proliferation and/or viability) was assessed by measuring the 3-(4.5-dimethylthiazol-2-yl)-2,5-diphenyl tetrazolium bromide (MTT, Sigma Aldrich, St. Louis, MO, USA) dye absorbance of the cells [26]. For this, a total of 5 × 10^4^ cells were seeded into 96-well plates containing 100 µL of the culture medium. The cells were treated with different concentrations of salamandrin-I (0, 1, 5, 10, 25, 50, and 100 µM) for 48 h. After this period, the cells were incubated with 10 µL of MTT (5 mg/mL) for 4 h. The supernatant was removed after centrifuging of the plates at 400× *g* for 10 min. The reaction product was solubilized by the addition of DMSO (Sigma Aldrich, St. Louis, MO, USA). The optical density was read on a Multiskan FC Microplate Photometer (Thermo Scientific, Waltham, MA, USA) at 570 nm. The IC_25_ and IC_50_ values were calculated using nonlinear regression analysis in GraphPad Prism 9 (GraphPad Software, Inc., San Diego, CA, USA).

### 2.3. Proliferation Assay

In order to determine whether salamandrin-I controls HL-60 proliferation, cells were stained with 7.5 μM of carboxyfluorescein succinimidyl ester (CFSE; Sigma Aldrich, St. Louis, MO, USA) and treated with IC_25_ and IC_50_ peptide doses for 48 h. Then, the cells were recovered and used to determine the percentage of CFSE+ cells with flow cytometry (FACSCalibur; BD Biosciences, East Rutherford, NJ, USA). Ten thousand events were recorded for each sample, and the data were analyzed using FlowJo software 10.0.7 (Treestar, Inc., Ashland, OR, USA).

### 2.4. Cell Cycle Analysis

A total of 1 × 10^6^ cells per well were seeded in 24-well plates and treated with IC_25_ and IC_50_ doses of salamandrin-I for 48 h. Then, the cells were fixed with 70% ethanol and stored at 4 °C for at least 2 h before analysis. For the flow cytometric analysis, the fixed cells were treated with 100 µg/mL of RNAse A for 10 min and then stained with 50 μg/mL of Propidium Iodide (PI) for 20 min. One hundred thousand events were collected using a FACSCalibur flow cytometer (BD Biosciences, East Rutherford, NJ, USA), and the data were analyzed using FlowJo software 10.0.7 (Treestar, Inc., Ashland, OR, USA).

### 2.5. Apoptosis Detection

The percentage of apoptotic cells was determined using the Annexin V/PI Apoptosis Detection Kit (BD Biosciences, East Rutherford, NJ, USA). For this, the HL-60 cell line was treated with IC_25_ and IC_50_ doses of salamandrin-I for 48 h. Then, the cells were recovered and stained with Annexin V and PI, following the manufacturer’s instructions. Ten thousand events were recorded from each sample with a FACSCalibur flow cytometer (BD Biosciences, East Rutherford, NJ, USA). The annexin V−/PI− cell population was considered viable, while the annexin V+/PI− and annexin V+/PI+ cell populations were considered apoptotic. The data were analyzed using FlowJo software 10.0.7 (Treestar, Inc., Ashland, OR, USA).

### 2.6. Determination of Lactate Dehydrogenase (LDH) Release

A total of 2 × 10^5^ cells were treated with IC_25_ and IC_50_ doses of salamandrin-I for 48 h. Then, supernatants from the cell cultures were collected and the LDH release measurement was performed using a CytoTox 96 Non-Radioactive Cytotoxicity Assay kit according to the manufacturer’s instructions (Promega Corp., Madison, WI, USA). Absorbance was determined using a Multiskan FC Microplate Photometer (Thermo Scientific, Waltham, MA, USA) at 490 nm.

### 2.7. Measurement of Mitochondrial Membrane Potential (ΔΨm)

To evaluate the mitochondrial membrane potential, 2 × 10^5^ cells were treated with IC_25_ and IC_50_ doses of salamandrin-I for 48 h. After this period, the cells were incubated with 5 µg/mL of rhodamine 123 for 20 min at room temperature. Then, the cells were washed with PBS and immediately analyzed with the FACSCalibur flow cytometer (BD Biosciences, East Rutherford, NJ, USA). Ten thousand events were acquired from each sample, and data analysis was performed using FlowJo software 10.0.7 (Treestar, Inc., Ashland, OR, USA).

### 2.8. Caspase-1 Inflammasome Assay

Caspase-1 activity was determined using a Caspase-Glo^®^1 inflammasome assay kit (Promega Corp., Madison, WI, USA). Briefly, 5 × 10^4^ cells were treated with an IC_50_ dose of salamandrin-I for 48 h. After incubation, 100 μL of the Caspase-Glo 1 reagent, with and without the caspase-1 inhibitor YVAD-CHO, was added to the plate and mixed using a plate shaker at 300 rpm for 30 s. The samples were incubated at room temperature, and luminescence was measured after 60 min using a Multimode Plate Reader (PerkinElmer, Waltham, MA, USA).

### 2.9. RNA Extraction, cDNA Synthesis, and Real-Time PCR

RNA extraction was performed using the TRI reagent (Sigma Aldrich, St. Louis, MO, USA) according to the manufacturer’s instructions. The amount and quality of the RNA samples were determined using Nanodrop One (Thermo Scientific, Waltham, MA, USA). One microgram of total RNA was converted to single-stranded cDNA using the High-Capacity cDNA Reverse Transcription Kit (Applied BioSystems, Waltham, MA, USA).

The PCR primers used to amplify the *CASP1*, *CASP3*, *CASP8*, *P53*, *TP73*, *BAX*, *BAK*, *NLRP1*, *NLRP3*, *GSDMD*, *IL-1β*, *CDK1*, *CDK2, KI-67*, and *GAPDH* genes are listed in Table 1. Real-time PCRs were performed using SYBR Green MasterMix (Thermo Scientific, Waltham, MA, USA) and a QuantStudio 1 Real-Time PCR System (Thermo Scientific, Waltham, MA, USA). All reactions were performed in technical duplicate, and the relative fold changes were obtained with the 2^−ΔΔCt^ method [27]. Median Ct values obtained from untreated cell lines were used as a reference.

### 2.10. Statistical Analysis

Data were tested for normal distribution with the Shapiro–Wilk normality test and analyses of skewness and kurtosis when applicable. All experiments were performed in triplicate, and values are expressed as means ± SEMs. All analyses were performed using Prism 9 software (GraphPad Software Inc., San Diego, CA, USA). The nonparametric Kruskal–Wallis test, followed by Dunn’s multiple comparison test, was used for numerical comparisons between groups. Probability values of *p* < 0.05 were accepted as indications of statistically significant differences.

## 3. Results

### 3.1. Salamandrin-I Treatment Decreases Human Leukemia Cell Viability

**Figure 1:**
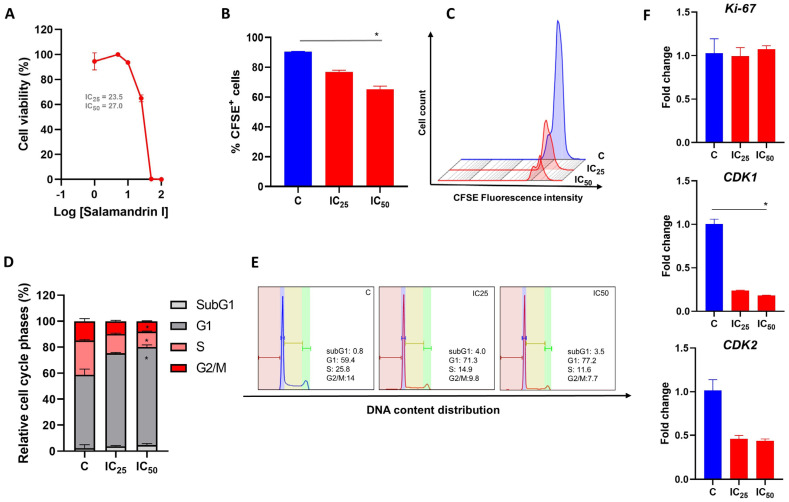
Antiproliferative effect of salamandrin-I. (**A**) HL-60 cells treated with increasing concentrations of salamandrin-I presented decreased viability, detected with an MTT assay. (**B**) Cell proliferation of HL-60 cells untreated (control, **C**) and treated with salamandrin-I at IC_25_ and IC_50_ doses. (**C**) Representative flow cytometry histogram showing CFSE^+^ in HL-60 cells treated with salamandrin-I at IC_25_ and IC_50_ doses. (**D**,**E**) Phases of the cell cycle, determined with flow cytometry, in C and HL-60 cells treated with salamandrin-I and exposed to IC_25_ and IC_50_ doses. (**F**) Expression of the genes *Ki-67*, *CDK*1, and *CDK2* in C and HL-60 cells treated with salamandrin-I at IC_25_ and IC_50_ doses. Real-time PCRs were performed in technical duplicate, and the relative fold change was obtained with the 2^−ΔΔCt^ method. Median Ct values obtained from untreated cell lines were used as a reference. Results are presented as means ± SEMs. * Asterisks indicate results that are statistically significant. * *p* < 0.05.

To determine whether salamandrin-I could compromise the viability of HL-60 cells, an MTT assay was performed. After 48 h of peptide treatment, the HL-60 cells showed a dose-dependent reduction in viability (Figure 1A). Nonlinear regression revealed that the IC_25_ value of the salamandrin-treated HL-60 cells was 23.5 µM and the corresponding IC_50_ value was 27 µM. Other myeloid leukemia cell lines were also evaluated and showed reduced cell viability after treatment with salamandrin-I (Supplementary Table S1).

### 3.2. Salamandrin-I Controls Cell Proliferation and Promotes Cell Cycle Arrest

The effect of salamandrin-I on HL-60 cell proliferation was determined after cell treatment with IC_25_ and IC_50_ doses for 48 h. Importantly, a reduction in the proliferation of the HL-60 lineage was observed with the IC_50_ dose (*p* = 0.02) and was accompanied by cell cycle arrest in the G1 phase (*p* = 0.02) as well as decreases in the percentages of cells in the S and G2/M phases (*p* = 0.02 and *p* = 0.02, respectively) (Figure 1B–E). In line, after the salamandrin-I treatment, the HL-60 cells showed significant reductions in their transcriptional levels of *CDK1* (*p* = 0.02) (Figure 1F).

### 3.3. Salamandrin-I Induces Cell Death in Human Leukemia Cell Line

**Figure 2:**
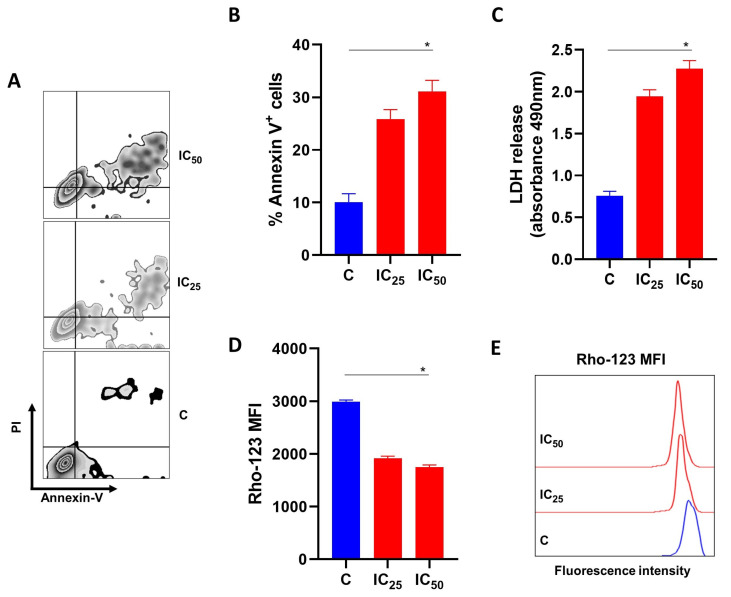
Effects of salamandrin-I on the viability and mitochondrial health of HL-60 cells. (**A**) Representative zebra plot showing the expressions of annexin-V and PI in C and HL-60 cells treated with salamandrin-I at IC_25_ and IC_50_ doses. (**B**) Annexin V^+^ HL-60 cells after treatment with salamandrin-I at IC_25_ and IC_50_ doses. (**C**) LDH releases in culture media for C and HL-60 cells treated with salamandrin-I at IC_25_ and IC_50_ doses. (**D**,**E**) Rhodamine 123 staining in C and HL-60 cells treated with salamandrin-I at IC_25_ and IC_50_ doses. Results are presented as means ± SEMs. Asterisks indicate results that are statistically significant. * *p* < 0.05.

To evaluate the mechanisms involved in the reduction in cell viability promoted by salamandrin-I, the HL-60 cells were treated with IC_25_ and IC_50_ doses of the peptide for 48 h and the percentage of cells that were positive for Annexin-V was determined with flow cytometry. Importantly, the treatment with salamandrin-I at the IC_50_ dose significantly promoted HL-60 cell death (*p* = 0.05) (Figure 2A,B).

### 3.4. Salamandrin-I Induces LDH Releases and Compromises the Mitochondrial Membrane Potential of Human Leukemia Cell Lines

After determining of the percentage of annexin-V-positive cells, the culture supernatant was collected to measure the LDH release. A significant increase in the LDH release was observed after the treatment of the HL-60 cell line with the salamandrin-I at the IC_50_ dose (*p* = 0.02) (Figure 2C). Importantly, the treatment with the salamandrin-I significantly impaired the mitochondrial membrane potential of the HL-60 cells (*p* = 0.02) (Figure 2D,E).

### 3.5. Salamandrin-I Activates Caspase-1 and Modulates Inflammasome Components in Human Leukemia Cell Line

**Figure 3:**
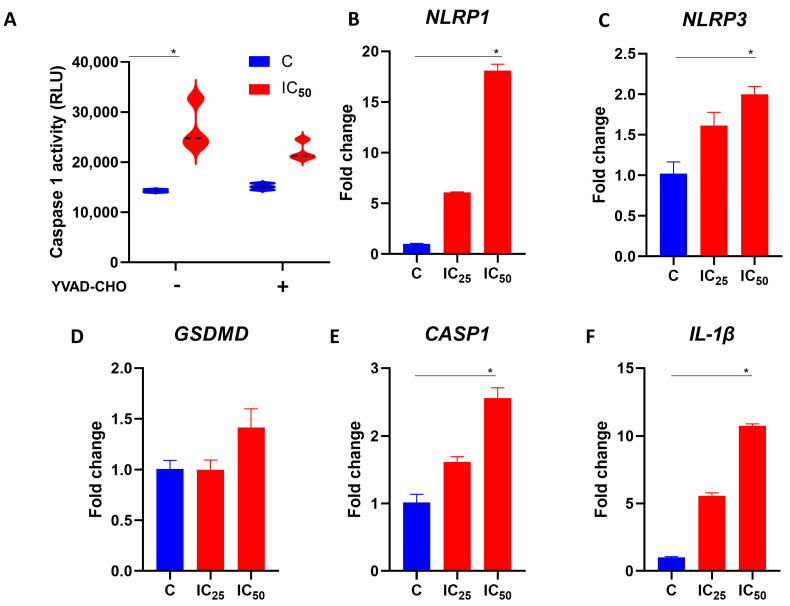
Modulation of inflammasome components with salamandrin-I. (**A**) Caspase 1 activity of C and HL-60 cells treated with salamandrin-I at IC_50_ dose. These experiments were conducted in the absence and presence of caspase 1 inhibitor YVAD-CHO. (**B**–**F**) Expression of the inflammasome components *NLRP-1*, *NLRP-3*, *GSDMD*, *CASP-1*, and *IL-1β*. Real-time PCRs were performed in technical duplicate, and the relative fold change was obtained with the 2^−ΔΔCt^ method. Median Ct values obtained from untreated cell lines (**C**, control) were used as a reference. Results are presented as means ± SEMs. * Asterisks indicate results that are statistically significant. * *p* < 0.05.

Using the Caspase-Glo 1 inflammasome assay kit, we evaluated whether salamandrin-I could modulate inflammasome components in HL-60 cells. Importantly, at the IC_50_ dose, there was an increase in caspase activity in the HL-60 cells (*p* = 0.02). Interestingly, in treatment with the caspase 1 inhibitor YVAD-CHO, no statistically significant changes were observed regarding caspase activity in the HL-60 cell line (Figure 3A). In line with the possible involvement of the inflammasome in the observed cell death process, the HL-60 cells showed elevated expressions of *NLRP1* (*p* = 0.02), *NLRP3* (*p* = 0.02), *CASP-1* (*p* = 0.02), and *IL-1β* (*p* = 0.02) after treatment with salamandrin-I (Figure 3B–F).

### 3.6. Salamandrin-I Increases Transcriptional Levels of CASP8 and Inhibits BCL-2 in HL-60 Cells

**Figure 4:**
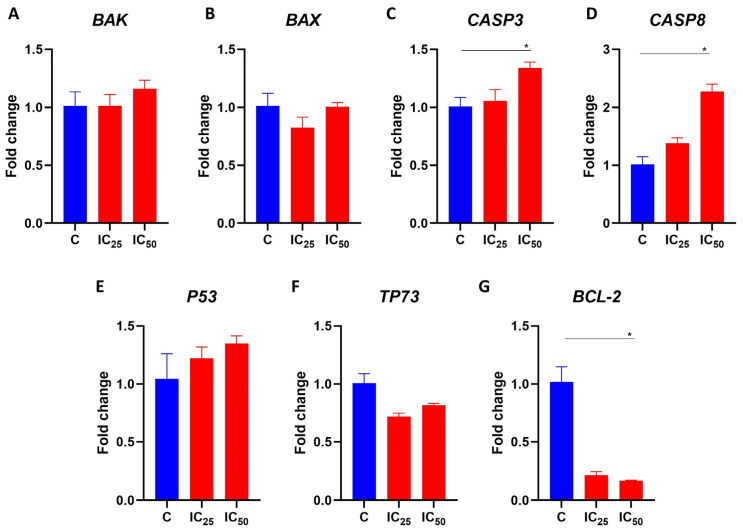
Gene expression analysis of selected transcripts. (**A**–**G**) Transcriptional levels of *BAK*, *BAX*, *CASP3*, *CASP8*, *P53*, *TP73*, and *BCL-2*. Real-time PCRs were performed in technical duplicate, and the relative fold change was obtained with the 2^−ΔΔCt^ method. Median Ct values obtained from untreated cell lines (**C**) were used as a reference. Results are presented as means ± SEMs. Asterisks indicate results that are statistically significant. * *p* < 0.05.

To better investigate the molecular effects that the salamandrin-I promoted in the HL-60 cells, these cells were cultured for 48 h with the peptide at the IC_25_ and IC_50_ doses, and their RNA was obtained for gene expression analysis. Real-time PCRs revealed that the transcript levels of *CASP8* (*p* = 0.03) and *CASP3* (*p* = 0.03) were significantly increased after the treatment of the HL-60 cells with the salamandrin-I at the IC_50_ dose. In addition, the HL-60 cells showed a lower expression of *BCL2* when treated with the salamandrin-I at the IC_50_ (*p* = 0.05) dose. No statistically significant changes were observed in the transcriptional levels of *BAK, BAX, P53,* or *TP73* (Figure 4).

## 4. Discussion

Along with cardiovascular disease, cancer is emerging as one of the leading causes of death worldwide. According to recent GLOBOCAN estimates produced by the International Agency for Research on Cancer, around 19 million new cases of cancer and approximately 10 million deaths have occurred annually in the world. Of this total, almost 475,000 new cases are leukemia, with approximately 300,000 deaths from this pathology being recorded [28]. The use of bioactive peptides has been explored as a promising approach for cancer therapy, especially for their selectivity, their high potency, and the possibility of reducing the toxic effects promoted by chemotherapy [29]. Here, we have evaluated for the first time the anticancer effect of the salamandrin-I peptide, using it in a cellular model of human myeloid leukemia, and we have demonstrated that in addition to controlling the growth of the HL-60 line, this peptide can efficiently promote its cell death.

Uncontrolled and independent proliferation of cancer cells is one of the main characteristics of tumors. Therefore, the development of new therapeutic approaches that can control tumor growth is of fundamental importance for cancer treatment [30]. The cell division process is controlled by several regulatory mechanisms to ensure the correct segregation of genetic material into new daughter cells. In eukaryotes, the cell cycle is composed of four phases, G1, S, G2, and M, each characterized by specific cellular activities and checkpoints. These checkpoints are crucial to verifying DNA integrity and activating mechanisms to either delay cell cycle progression or initiate programmed cell death. DNA replication happens during the S phase, while genetic material segregation is divided into two new daughter cells during the M phase. The G1 and G2 phases act as decision points for the cell’s commitment to DNA replication and cell division. In cancer cells, some of these aspects are disrupted, enabling the cells to escape repair mechanisms and undergo uncontrolled proliferation [31,32]. Interestingly, salamandrin-I was able to significantly control HL-60 proliferation in a dose-dependent manner. After salamandrin-I treatment, a high percentage of HL-60 cells remained in the G1 phase of the cell cycle, and there were reductions in the numbers of cells in the S and G2/M phases. Cell cycle progression is driven by cyclin-dependent kinases (CDKs), which bind to different cyclins and allow coordinated cell cycle progression. However, it seems that only some complexes can regulate the cell cycle [33]. CDK1 and CDK2 are essential for controlling the cell cycle, acting in the G1-S and G2-M phase transitions of eukaryotic cells [34,35]. The complex formed of cyclin E and CDK2 mediates the initiation of the S phase and DNA synthesis. At the end of the S phase, cyclin A forms a complex with CDK2, enabling the transition to the G2 phase. A subsequent complex formed of cyclin A and CDK1 leads cells to the M phase [36,37]. Corroborating the functional tests carried out, the salamandrin-I drastically inhibited the *CDK1* and *CDK2* transcriptional levels in the HL-60 cells.

The identification of antioxidant peptides with important anticancer properties has been reported in the literature [38,39]. Interestingly, it was previously demonstrated that at up to a 100 µM concentration, salamandrin-I was not toxic when cultured with human microglia and red blood cells [7]. However, using an in vitro model of human leukemia, we identified that in addition to controlling cell division, salamandrin-I can induce apoptosis of the HL-60 cell line, which is accompanied by greater LDH release and loss of mitochondrial transmembrane potential. In addition, the treated cells also showed increased transcriptional levels of *CASP-3* and *CASP-8* and decreased levels of antiapoptotic factor *BCL-2.* The BCL-2 protein family includes both proapoptotic and antiapoptotic members, which play an important role in cell survival by modulating the mitochondrial pathway of apoptosis [40,41]. This intrinsic pathway of apoptosis is triggered by a variety of stress signals, leading to BAX and BAK activation and subsequent damage of the mitochondrial outer membrane (MOMP) [42]. This disrupted membrane allows the release of several apoptotic factors, including cytochrome c, that are able to activate members of the caspase family [43,44]. Initiator caspase-9 is activated by conformational changes induced by apoptosomes and further processes the downstreams of some effector caspases, such as CASP-3 and CASP-7, leading to apoptosis [45,46]. BCL-2 and BCL-xL are able to counter these proapoptotic events by binding and sequestering activated BAX and BAK proteins [47]. Despite the observation of a decrease in the mitochondrial membrane potential in HL-60 cells after treatment with this peptide, we did not observe changes in the *BAX* or *BAK* expressions. At the molecular level, we noticed that salamandrin-I treatment provoked a trend of *P53* increase in HL-60 cells, although this difference was not statistically significant.

With the purpose of advancing the identification of mechanisms associated with cell death promoted by salamandrin-I, we evaluated whether this peptide could modulate components involved in pyroptosis, an inflammatory programmed cell death pathway. Pyroptosis is a caspase-1-dependent mechanism that leads to cell swelling, membrane pore formation, and plasma membrane rupture [48]. Caspase-1 activation induces gasdermin D (GSDMD) cleavage and activation, leading to the oligomerization of the fragments into the plasma membrane, forming pores. Additionally, caspase-1 is involved in the subsequent proteolytic cleavage of the IL-1β and IL-18 precursor forms. After membrane rupture, these inflammatory cytokines and several cytosolic contents are released into extracellular space, characterizing the inflammatory potential of pyroptosis [48,49,50,51].

The canonical pyroptosis pathway is mediated by inflammasome assembly. An inflammasome is a multiprotein signaling complex responsible for controlling and coordinating inflammatory responses in the presence of pathogens and signs of damage to the cell or cellular structures such as the mitochondria [52,53]. NLRP3, a member of the nucleotide-binding domain (NOD) receptor family, is one of the receptor proteins responsible for inflammasome formation, which responds to several types of stimuli and signals that are related to PAMP and DAMP as well as being related to the mechanisms of pyroptosis and mitochondrial damage [53,54,55]. Interestingly, salamandrin-I-treated HL-60 cells presented significantly increased levels of *NLRP-1*, *NLRP-3*, *CASP-1,* and *IL-1β* transcripts, as well as caspase 1 activity, suggesting that the cell death observed was influenced by inflammasome assembly [56]. Importantly, it has been reported that inflammasome activation can be triggered by CASP-8 and mitochondrial injury [57,58,59], which is in line with our results.

This is the first demonstration of the anticancer role of salamandrin-I. These results are important, as they offer important insights into the field of anticancer therapy and allow the design of future bioactive molecules using salamandrin-I as a template.

## Figures and Tables

**Table 1:** qPCR primer sequences.

Gene	Primer	Sequence (5′-3′)
*GAPDH*	Forward	TCAACGACCACTTTGTCAAGCTCAGCT
	Reverse	GGTGGTCCAGGGGTCTTAC
*CASP1*	Forward	AAGACCCGAGCTTTGATTGACTC
	Reverse	AAATCTCTGCCGACTTTTGTTTCC
*CASP3*	Forward	CTAGCGGATGGGTGCTATTG
	Reverse	GATACACAGCCACAGGTATGAG
*CASP8*	Forward	GGATGGCCACTGTGAATAACTG
	Reverse	TCGAGGACATCGCTCTCTCA
*P53*	Forward	AGAAAACCTACCAGGGCAGC
	Reverse	ACATCTTGTTGAGGGCAGGG
*TP73*	Forward	GGAAGATGGCCCAGTCCAC
	Reverse	GGGAAGGTCGAAGTAGGTGC
*BAX*	Forward	CAGACCGTGACCATCTTTGT
	Reverse	GCCTCAGCCCATCTTCTTC
*BAK*	Forward	GTTTTCCGCAGCTACGTTTTT
	Reverse	GCAGAGGTAAGGTGACCATCTC
*NLRP1*	Forward	AAGGGGCAGGCCACTCTCCCTC
	Reverse	TGAGGCAGAGATTTCTCTCCAG
*NLRP3*	Forward	TCCTCGGTACTCAGCACTAATCAG
	Reverse	GGTCGCCCAGGTCATTGTTG
*GSDMD*	Forward	ATGAGGTGCCTCCACAACTTCC
	Reverse	CCAGTTCCTTGGAGATGGTCTC
*IL-1β*	Forward	AGAAGTACCTGAGCTCGCCA
	Reverse	TGTTTAGGGCCATCAGCTTCA
*CDK1*	Forward	CTTGGCTTCAAAGCTGGCTC
	Reverse	GGGTATGGTAGATCCCGGCT
*CDK2*	Forward	CCAGGAGTTACTTCTATGCCTGA
	Reverse	TTCATCCAGGGGAGGTACAAC
*KI-67*	Forward	TAACACCATCAGCAGGGAAAG
	Reverse	CTGCACTGGAGTTCCCATAAA

*GAPDH*: Glyceraldehyde-3-Phosphate Dehydrogenase; *CASP1*: Caspase 1; *CASP3*: Caspase 3; *CASP8*: Caspase 8; *P53*: Tumor Protein P53; *TP73*: Tumor Protein P73; *BAX*: BCL2 Associated X, Apoptosis Regulator; *BAK*: BCL2 Antagonist/Killer 1; *NLRP1*: NLR Family Pyrin Domain Containing 1; *NLRP3*: NLR Family Pyrin Domain Containing 3; *GSDMD*: Gasdermin D; *IL-1β*: Interleukin 1 Beta; *CDK1*: Cyclin Dependent Kinase 1; *CDK2*: Cyclin Dependent Kinase 2.

## Data Availability

The authors confirm that all data underlying these findings are fully available.

## Supplementary Materials

The following supporting information can be downloaded at: https://www.mdpi.com/article/10.3390/pharmaceutics15071864/s1, Figure S1: MS/MS spectrum of synthetic salamandrin-I; Table S1: IC25 and IC50 values for salamandrin-I in human myeloid cell lines.
