# Supplementary material for: The Peptide Salamandrin-I Modulates Components Involved in Pyroptosis and Induces Cell Death in Human Leukemia Cell Line HL-60
Source: Pharmaceutics. 2023 Jul 1;15(7):1864. doi: 10.3390/pharmaceutics15071864 (PMC10384876; doi:10.3390/pharmaceutics15071864)
Supplement: Supplementary file 1 [file pharmaceutics-15-01864-s001.zip › pharmaceutics-2411270-supplementary.pdf]

Supplementary table S1. IC_{25} and IC_{50} values for salamandrin-I in human myeloid cell lines

	HL-60	HEL	K562
Salamandrin I (μM)			
IC25	23.5	21.6	13.8
IC50	27.0	26.2	31.3

HL-60: LMA M2 cell line; HEL: LMA M6 cell line; K562: chronic myeloid leucemia cell line

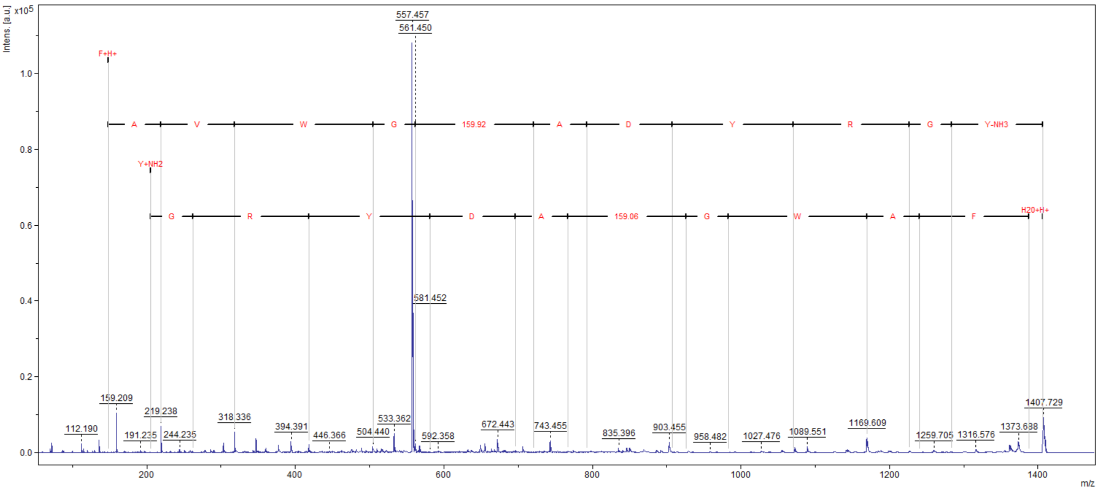

Figure S1. MS/MS spectrum of synthetic salamandrin-I, $[M + H]^+ = 1406.6$ Da; amino acid sequence determined: FAVWGCADYRGY-NH_2.
